# Chemical content and estimated sources of fine fraction of particulate matter collected in Krakow

**DOI:** 10.1007/s11869-016-0407-2

**Published:** 2016-05-10

**Authors:** Lucyna Samek, Zdzislaw Stegowski, Leszek Furman, Joanna Fiedor

**Affiliations:** Faculty of Physics and Applied Computer Science, AGH University of Science and Technology, 30 Mickiewicza Ave., 30-059 Krakow, Poland

**Keywords:** Particulate matter, Energy dispersive x-ray spectrometry, Positive matrix factorization

## Abstract

The monitored level of pollution remains high in Krakow, Poland. Alerts regarding increased levels of pollution, which advise asthmatics, the elderly, and children to limit their exposure to open air, continue to be issued on numerous days. In this work, seasonal variations in PM2.5 (particulate matter containing particles with aerodynamic diameter no higher than 2.5 μm) concentrations are shown. An increasing trend is reported, which is enhanced during the colder seasons. The mean PM2.5 concentrations in Krakow exceeded the target value of 25 μg/m^3^ specified for 2015 in the spring, autumn, and winter seasons. For this reason, particulate matter pollution is of special concern. Elemental concentrations as well as the presence of black carbon (BC) and black smoke (BS) in PM2.5 samples were determined. Seasonal variations of Cl, K, Ca, Ti, Mn, Fe, Cu, Zn, Br, Rb, Sr, and Pb concentrations were observed whereas V, Cr, Ni, BC, and BS concentrations did not significantly change with the time of year. Seven factors were identified by the positive matrix factorization (PMF) technique, and one was non-identified. They were attributed to the following sources of pollution: steel industry, traffic (diesel exhaust), traffic (gasoline exhaust, brake wear), road dust, construction dust, combustion (biomass, coal), and non-ferrous metallurgical industry. The last, non-identified source, could be attributed to secondary aerosols. It is worth to mention that combustion shows significant seasonal variations with a high impact in winter. The reported results of the completed studies may significantly aid in solving air quality issues in the city by highlighting major sources of air pollution.

## Introduction

According to European Directives, the concentrations of NO_x_, CO_2_, and particulate matter significantly exceed the specified limit values (Ostro et al. 2015; Kim et al. 2004; Lim et al. 2011). Increased levels of air pollution can negatively affect human health, especially prolonged exposure to polluted air may cause respiratory and cardiovascular diseases. The overall mortality and morbidity can also be influenced (Brunekreef and Holgate 2002; Anenberg et al. 2010; Samek 2016). A component of air pollution which is given special attention is PM_2.5_—a number of research groups has reported major and trace elements concentrations in PM_2.5_ (Cuccia et al. 2013; Yu et al. 2013; Moreno et al. 2006; Samek et al. 2015, 2016; Zhang et al. 2015, Terrouche et al. 2016). Due to the fact that elemental composition is determined by unique factors, its determination may lead to the identification of sources of air pollution. Properly identified sources can then improve the efforts to minimize pollution levels in different cities. Since chemical content of air particulate matter determines its toxicity, receptor models are used for source identification and apportionment based on the concentrations of chemical species in PM_2.5_ (Mazzei et al. 2008; Laupsa et al. 2009; Querol et al. 2007; Masiol et al. 2014).

Krakow is located in a valley in Southern Poland. The city is characterized by a high level of particulate matter pollution. Steel and non-ferrous metallurgical industries are located within the city. Other sources of PM pollution are also present, they include traffic (from diesel or gasoline exhausts), brake, and tire wear. Additionally, the air in Krakow is influenced by long-range transport of aerosols (e.g., secondary aerosols) (Samek 2012; Samek et al. In press). These studies identified potential sources. However, the time series of source contributions was not identified in these studies due to limited number of samples, short duration of sampling, or discontinuous sampling. A long period of daily PM_2.5_ data is needed in order to obtain a precise source identification. The present study was designed to fill this gap.

The aim of this work is to present results of chemical analyses of PM_2.5_ samples collected during a 1-year period (2014/2015) in an urban area of Krakow. Major and trace element, black carbon, and black smoke concentrations as well as seasonal variations were determined. Additionally, source identification was performed and seasonal variations of likely PM_2.5_ sources were unveiled. The application of a full year continuous data could not only improve the efficiency of positive matrix factorization (PMF) analysis, but also help to perform the time series analysis of various sources.

In this work, PMF is applied as described by Paatero (Paatero 1997). The sources are classified as natural or anthropogenic. Natural sources include suspended soil and road dust, sea salt, forest fires as well as long-range transported dust (Marconi et al. 2014). The following are a part of anthropogenic sources: industry, traffic, combustion of biomass, or coal for residential heating.

## Experimental

### Sampling

Samples were collected by the Voivodship Inspectorate of Environmental Protection in Krakow. The site selected for the study was an urban area of Krakow, specifically, the southeastern part of the city (district Kurdwanow). The major local sources of pollution are municipal emissions, industry, and traffic. Traffic in the city is dense with frequent traffic jams. Factories are located at a distance of about 10 km from the sampling site. Additionally, a power plant is located in the southern area of the city. The Upper Silesian industry area can be found approximately 80 km to the west from Krakow. Moreover, the zinc industry is situated about 50 km to the north of the city. Twenty-four-hour PM_2.5_ fraction samples were collected between February 1, 2014 and January 31, 2015 with the use of a low volume sampler with a flow rate of 2.3 m^3^/h. Quartz (46.2 mm) filters were used as a support. Overall, 194 samples were collected during the entire year.

### Chemical analysis

PM_2.5_ concentrations were determined by the Voivodship Inspectorate of Environmental Protection in Krakow. Concentrations of the following elements were quantified: Cl, K, Ca, Ti, V, Cr, Mn, Fe, Ni, Cu, Zn, Br, Sr, Rb, and Pb. Samples of PM_2.5_ were analyzed with the use of a multifunctional energy dispersive X-ray fluorescence spectrometer as thin samples. The instrument is a micro-beam X-ray fluorescence spectrometer with capillary X-ray optics, a broad X-ray beam from a molybdenum secondary target for XRF analysis of bulk samples, and a total reflection X-ray technique. The molybdenum tube is the source of X-rays. The tube has the power of 2 kW. The excited X-rays were detected by a Si(Li) detector with resolution of 170 eV at an energy of 5.9 keV. Data collection was completed using the Canberra system. The measurements were carried out under the following conditions: voltage of 55 kV, current of 30 mA, measurement time of 10,000 s, and under atmospheric air. In order to calculate the concentrations of different elements in the filters, the spectrometer was calibrated using thin-film standards (Micromatter, USA). The calibration was verified by the analysis of the NIST Standard Reference Material (2783-Air particulate matter on filter media). Table 1 presents certified and measured elemental concentrations of the NIST Standard Reference Material. The XRF spectra were quantitatively analyzed with the use of the QXAS package (Vekemans et al. 1994).

**Table 1:** Measured and certified concentrations of elements in NIST SRM2783

Element	Measured values (ng)	Certified values (ng)
K	6011 ± 4500	5280 ± 520
Ca	18,780 ± 3500	13,200 ± 1700
Ti	1384 ± 130	1490 ± 240
Cr	115 ± 67	135 ± 25
Mn	319 ± 66	320 ± 12
Fe	27,111 ± 678	26,500 ± 1600
Cu	398 ± 25	404 ± 42
Zn	2077 ± 63	1790 ± 130
Pb	244 ± 25	317 ± 54

The presence of black carbon (BC) and black smoke (BS) was determined by UV-VIS spectroscopy. Spectroscopic measurements were performed using a Varian Cary 50-Bio UV-VIS spectrophotometer (Agilent). Transmittance was recorded at the 880 nm wavelength, triplicate for each sample. Additionally, transmission spectra were collected in the range of 200–1000 nm. All measurements were carried out in reference to air. Concentrations of BC and BS were calculated according to formulas listed by Quincey P. (Quincey 2007).

### Statistical analysis

Source apportionment analysis was carried out using the positive matrix factorization receptor model (version PMF5.0) developed by the United States Environmental Protection Agency (US EPA).

PMF requires two inputs to run, namely concentration and its uncertainty. In this work, if the concentration was less than or equal to the detection limit (DL) provided, the uncertainty was calculated as five sixths DL and the concentration as one half DL (Pollisar 1998). Missing data was substituted with median values, and the corresponding uncertainties were replaced by four times the median values. PM_2.5_ concentration was included as a total variable, and all the species were characterized as “strong,” “weak,” or “bad” depending on the signal to noise ratio.

The input data for the PMF model included 194 samples with 18 species (concentration of PM_2.5_, 15 elements, BC and BS). Concentrations of three elements, V, Sr, and Rb, were excluded because many of the corresponding values were below the detection limit and they were characterized by low signal to noise values.

## Results and discussion

Table 2 shows concentrations of PM_2.5_, BC, BS, and major and trace elements in PM_2.5_ during different seasons of the 2014/2015 period. The lowest concentration of PM_2.5_ was observed in the summer. It was significantly below the target value specified by the EU Directive (2008). Concentrations reported during spring and autumn were slightly higher than the target value whereas those observed in the winter were as high as twice the target value. In Poland, in the Regulation of the Minister of the Environment (2012), the limit value of PM_2.5_ concentration was 25 μg/m^3^—the same as target value to be met in 2015 (phase I) as well as target value equal to 20 μg/m^3^ to be met in 2020 (phase II). Strong seasonal variations were noted for concentrations of Cl, K, Br, Pb, Cu, and Zn. Cl and K can originate from combustion of coal and/or biomass. Ratios of Cu to Zn were in the range of 0.09–0.16 depending on the season. A Cu to Zn ratio equal to 0.3 indicative of traffic was reported by (Mazzei et al. 2008). Ratios of Cu to Pb were 0.43 and 0.46 in spring and summer, respectively. A higher value of 0.69 was reported in the summer, and a low number of 0.28 was found in the winter. These elements present in ratios in the range of 2.3–3.0 were identified as indicators of traffic by (Mazzei et al. 2008). These results suggest that a source of Pb other than traffic also exists. The Zn to Pb ratio in winter was equal to 2.74 and in other seasons of the year it was in the range 3.28–3.35. The ratio of Pb to Br was equal to 1.64–1.85, and it remained constant during all seasons of the year. Concentrations of Ca and Ti were the highest in spring and the lowest in autumn. These elements can be related to construction dust created at prevalent construction sites in Krakow. Other elements such as V were present in constant concentrations during the year. Ni concentrations in spring and summer were twice as large as those reported in winter and autumn. V and Ni are indicators of traffic. Black carbon and black smoke had the highest values in autumn and were higher than those found in Ghent and Amsterdam but were similar to those noted in Barcelona. During the remaining seasons, the values were comparable between the various cities. Based on the assumption that black carbon is a tracer for primary emissions, mostly derived from traffic, these results suggest that the influence of traffic on air pollution is higher in Krakow than in Amsterdam and Ghent and on a similar level as in Barcelona (Viana et al. 2007).

**Table 2:** Concentrations of chemical species in Krakow during different seasons of the year (concentrations of PM_2.5_, BC, BS are in μg/m^3^, and rest of species in ng/m^3^)

Element	Spring	Summer	Autumn	Winter
PM_2.5_	31 ± 23	12.7 ± 4.6	30 ± 21	57 ± 39
Cl	674 ± 1408	<DL	361 ± 704	3609 ± 3548
K	255 ± 411	17 ± 36	49 ± 115	507 ± 537
Ca	264 ± 380	59 ± 144	36 ± 72	169 ± 221
Ti	13 ± 13	8.4 ± 7.5	6.8 ± 5.3	7.7 ± 7.3
V	1.7 ± 1.3	1.7 ± 1.6	1.7 ± 1.3	1.6 ± 1.3
Cr	3.8 ± 2.7	3 ± 2	6.5 ± 5.1	6.0 ± 4.4
Mn	5.9 ± 4.5	3.5 ± 2.4	5.1 ± 4.7	7.7 ± 8.7
Fe	261 ± 217	102 ± 90	195 ± 139	267 ± 219
Ni	1.9 ± 1.0	1.7 ± 1.0	0.9 ± 0.8	0.6 ± 0.2
Cu	8.5 ± 9.1	2.9 ± 2.2	5.5 ± 4.6	12 ± 11
Zn	67 ± 52	18 ± 10	59 ± 55	118 ± 89
Br	11 ± 9	3.3 ± 1.5	9.8 ± 8.5	25 ± 17
Rb	1.5 ± 1.0	0.9 ± 0.5	1.4 ± 1.1	2.3 ± 1.3
Sr	1.9 ± 1.1	1.7 ± 1.1	1.1 ± 0.8	0.9 ± 0.9
Pb	20 ± 17	5.4 ± 3.8	18 ± 16	43 ± 32
BC	2.4 ± 1.3	2.7 ± 1.2	2.8 ± 1.2	2.6 ± 1.3
BS	10.4 ± 6.5	12.0 ± 5.6	12.6 ± 6.2	11.4 ± 6.1

St.dev.—variability of concentrations during the measured period

**Table 3:** Factors with attributed sources and their contribution to PM mass concentrations

Factor number	Attributed source	Indicators	Source contributions (%)			
			Spring	Summer	Autumn	Winter
Factor 1	Steel industry	Mn, Fe, Zn, Br, Pb	13	11.7	7	3.3
Factor 2	Traffic (diesel exhaust)	Ni, BC, BS	6.2	24.9	9.5	15.7
Factor 3	Traffic (gasoline exhaust)	Cr, Cu, Br, BC,BS	33.7	35	39	17.8
Factor 4	Road dust	Cu	4.0	3.2	2.2	2.3
Factor 5	Construction dust and/or soil dust	Ca, Ti, Cr, Mn, Fe, Ni	10.8	13	8.5	10
Factor 6	Combustion coal and/or biomass	Cl, K,	5	0.3	0.9	24.6
Factor 7	Non-ferrous metallurgical industry	Zn, Br, Pb	15.7	11	18	18
Factor 8	Non-identified source		11.2	5	9.9	2.4

**Fig. 1:**
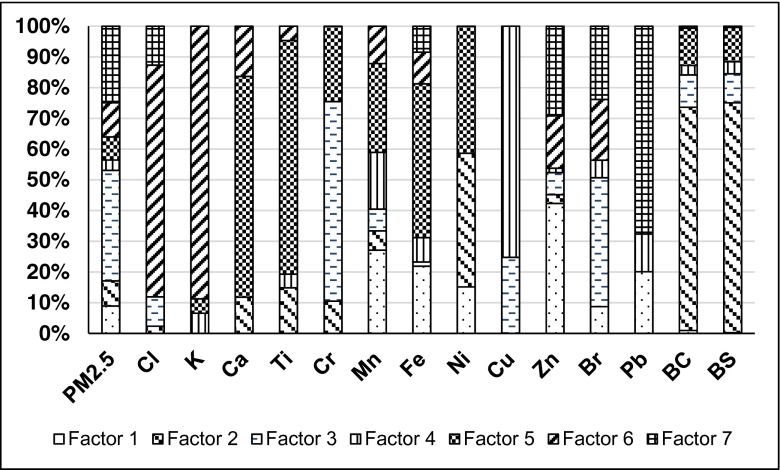
Factor profiles of fine particulate matter. For attributions to factors, see Table 3

**Fig. 2:**
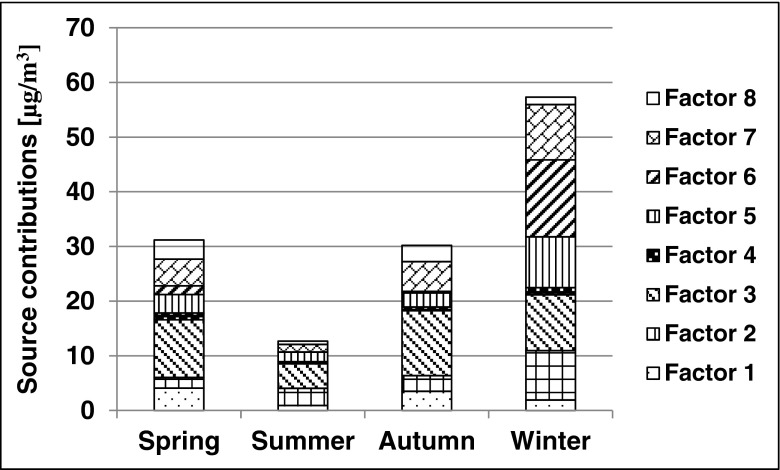
Source contributions in micrograms per cubic meter. For attributions to factors, see Table 3

**Fig. 3:**
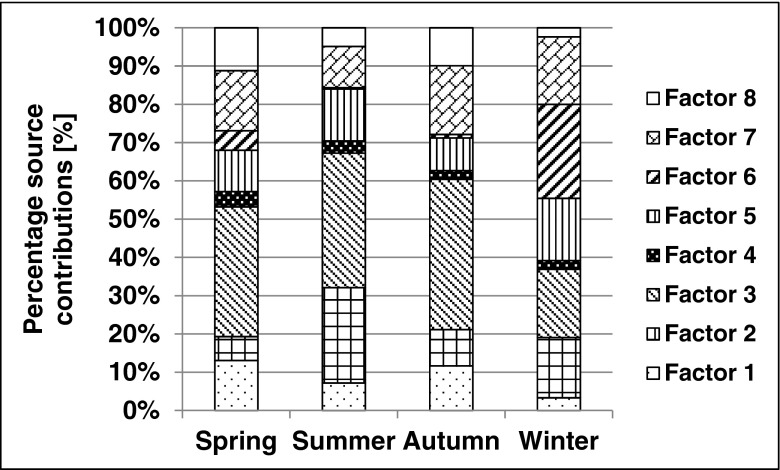
Percentage source contributions

Figure 1 presents the factor profiles. Figure 2 and Fig. 3 show source contributions in microgams per cubic meter and percent, respectively. A positive matrix factorization model was used for source identification and apportionment. Seven factors were established, and one was non-identified. Table 3 shows condensed data of contributions of each source and factors with attributable indicators. Mazzei et al. (2008) found indicators of the steel industry as Fe, Mn as well as traffic Cu, Zn, and Pb. Indicators of the non-ferrous metallurgical industry can be Cu, Zn, Pb, As, Cr, Ni, and Co while burning of oil Ba, Co, Ni, V, Cr, and Mn (Kabata-Pendias, Pendias 1999). Jedynska et al (Jedynska et al. 2014) reported that EC can be an indicator of diesel emissions. Yu et al. (2013) published that biomass burning indicator was K as well as fossil fuel combustion indicators were Cl, V, Ni, As, and Pb. Confirmation of this finding also is the paper of Viana (Viana et al. 2008).

In a previous study (Samek et al. In press), fine fraction principal component analysis (PCA) and multilinear regression analysis (MLRA) were performed. The contribution of municipal emissions combined with industry was estimated to be 49.2 % during winter, that of traffic was at 37.8 %. In the present study, the contribution of traffic in winter was similar at 36.5 % (15.7 % diesel exhaust, 17.8 % gasoline exhaust, 3 % road dust) while that of combustion was equal to 25 %; the steel industry contributed 3.4 % and the non-ferrous metallurgical industry added 18 %. The previously mentioned study (Samek et al. In press) reported a mean contribution of traffic equal to 53 % (40–60 %) in summer. In this study, it was found to be equal to 62.9 % (24.9 % diesel exhaust, 35 % gasoline exhaust, 3 % road dust). The cited work stated the contribution of industry in summer to be equal to 18 % (in the range of 5–40 %). In this study, the corresponding contribution was at 20.3 % (3.3 % steel industry, 17 % non-ferrous metallurgical industry). PMF results were consistent with the previously completed research. This research provided an increased number of factors indicated as pollutants as well as a more complete analysis than previous ones. This study complements previous investigations performed during a more limited time period (1 month in summer and one in winter) by delivering new valuable information.

## Conclusions

Chemical characterization of PM_2.5_ fraction collected in Krakow was reported. Certain elements such as Cl, K, Br, Pb, Cu, and Zn show strong seasonal variations of concentrations whereas others—V, Ni, BC, BS—do not exhibit such deviations. Eight contributing factors were identified, and a single pollution source was attributed to each one. Strong seasonal variations were associated with combustion, with the peak value reported in winter. Contributions of traffic were constant throughout the year. This study is consistent with a previously published work regarding PM_2.5_. It involved an extended period of time, a larger number of analyzed samples, and, as a result, more detailed information. This data is particularly relevant to the identification of pollution sources in Krakow. However, further analyses need to be performed in order to determine the impact of the described pollution on human health.
